# Quantum-Inspired Fast Algorithm and Circuit Realization for Constrained Combinatorial Optimization Problem

**DOI:** 10.34133/research.1345

**Published:** 2026-06-29

**Authors:** Haosen Chen, Shailan Deng, Tian Chen, Xiangdong Zhang

**Affiliations:** Key Laboratory of Advanced Optoelectronic Quantum Architecture and Measurements of Ministry of Education, School of Physics, Beijing Institute of Technology, Beijing 100081, China.

## Abstract

Constrained combinatorial optimization (CCO) problems are prevalent across various fields and represent key challenges in computational science and engineering. Although numerous classical and quantum algorithms have been proposed to tackle these problems, substantial limitations still persist. Classical algorithms exhibit exponential computational complexity growth with scale and persistent vulnerability to local minima traps. Quantum computing, while offering theoretical advantages through global superposition, faces practical barriers such as short decoherence times and current hardware limitations. To address these challenges, we propose a quantum-inspired fast algorithm for solving CCO problems. Our approach enhances global search capability with superposition encoding and avoids constraint-induced local minima​​ via a project–feedback strategy. Particularly, our method aligns with mature electronics manufacturing and demonstrates a proof-of-concept implementation in classical systems, indicating high-efficiency potential for solving constrained optimization problems.

## Introduction

Constrained combinatorial optimization (CCO) problems involve finding the optimal configuration satisfying given constraints from a discrete solution space. These problems represent core challenges in computational science and engineering optimization [1,2]. With the surging demand for efficient decision-making in fields like logistics scheduling, chip design, and artificial intelligence, the computational efficiency and stability of solving CCO have been a critical bottleneck in contemporary industrial applications. At their core, CCO problems feature extremely high-dimensional discrete landscapes shaped by the interplay between objective functions and constraint-induced feasibility regions, making efficient navigation of these landscapes a central challenge for both algorithmic and physical computing paradigms.

To overcome these challenges, numerous classical approaches have been proposed. Classical heuristic algorithms [3–7] [e.g., simulated annealing (SA), ant colony optimization (AC), and genetic algorithm] alleviate computational demands through stochastic search and local optimization strategies. While effective in moderate-scale scenarios, these methods often struggle to balance global exploration with rapid convergence, leading to either premature trapping in local minima or increased computational overhead. In parallel, continuous relaxation-based frameworks improve tractability by embedding discrete decision variables into continuous spaces and optimizing a surrogate objective defined over the relaxed domain [8,9]. Such approaches are widely adopted due to their flexibility and compatibility with gradient-based optimization techniques. In these formulations, the search process is primarily guided by the minimization of a fixed relaxed objective, where the geometry of the surrogate landscape determines the optimization trajectory.

Beyond purely algorithmic approaches, recent years have witnessed the emergence of physics-assisted hardware solvers, such as Ising machines implemented via compute-in-memory architectures [10–12] and photonic or electronic oscillator networks [13–19]. By exploiting the intrinsic dynamics of physical systems to perform large-scale parallel state evolution, these platforms offer a promising pathway to alleviate the memory and power limitations of conventional digital computing. Despite rapid progress, practical implementations remain constrained by device noise, parameter variability, and limited programmability, which can hinder solution stability and scalability, particularly in problems involving complex constraints.

Meanwhile, quantum computing offers theoretical potential for efficiently escaping local optima by leveraging the global superposition inherent in quantum systems [20–27]. For instance, quantum annealing searches for the global optimum through adiabatic evolution and has demonstrated promising efficiency in solving low-dimensional sparse problems [28–31]. Nevertheless, its applicability to large-scale real-world applications remains limited due to short decoherence times and constraints of sparse topology mapping. In response, several quantum-inspired algorithms have been developed in recent years to solve combinatorial optimization problems while avoiding the hardware constraints of quantum devices [11,32–35]. However, a common drawback among these methods is the lack of effective mechanisms for handling constraints, ​​which often leads to infeasible solutions or substantially degraded optimization quality in tightly constrained scenarios. Consequently, developing a unified framework that can integrate scalable computation, robust optimization dynamics, and effective constraint handling remains an important open challenge in solving CCO problems.

In this paper, we propose a quantum-inspired fast algorithm (QIFA) for solving CCO problems. QIFA formulates the search as a quantum-inspired evolution of analog state variables that emulate superposition-like exploration, while a projection–feedback mechanism is intrinsically combined with the evolution to enforce feasibility throughout the search. In this way, exploration and constraint satisfaction co-evolve, establishing a unified dynamical optimization framework in which feasibility and search dynamics remain tightly coupled. Using the Traveling Salesman Problem (TSP) as a benchmark task, we compare the solution performance of our algorithm against various other methods at different scales, demonstrating its high efficiency and superior solving capability. For the tested TSP instances, the algorithm empirically exhibits an approximately linear relationship between city size and the number of iterations required to reach near-optimal solutions. Furthermore, we design and implement the algorithm in analog circuitry. Experimental validation on a 20-city global TSP benchmark confirms the feasibility and effectiveness of our analog circuitry hardware in real-world optimization scenarios, without suffering from inherent quantum limitations such as decoherence or cryogenic requirements. We also discuss the theoretical solving time achievable with current cutting-edge experimental platform technology. Overall, this work provides a novel design paradigm for developing hardware-friendly and scalable constrained optimization strategies.

## Results

### Theory of QIFA for CCO problems

We consider CCO problems with first-order constraints and discrete variables, typical examples of which include TSP, Knapsack Problem (KP), and Job-shop Scheduling Problem (JSP). Such problems are characterized by 3 key elements: a set of discrete decision variables x, an objective function $f\left(x\right)$, and a set of constraints $\left\{g_{k}\left(x\right)|\;k\in \left\{1,\;\ldots ,\;K\right\}\right\}$, where k indexes the constraints and K denotes their total number. The optimization task is to minimize the objective function $f\left(x\right)$ over the variables x while satisfying all constraints $g_{k}\left(x\right)=0$.

**Fig. 1.**
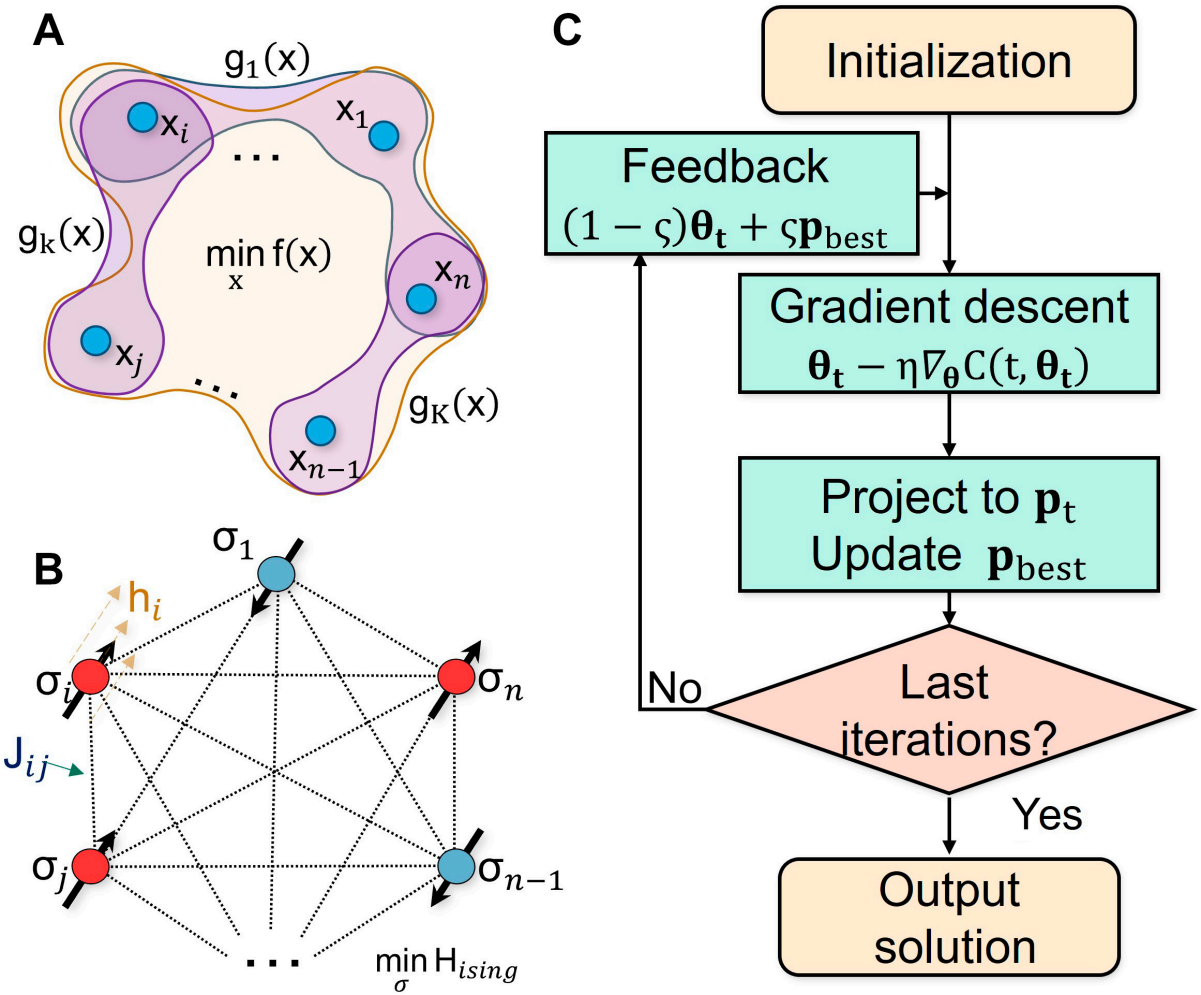
Framework of QIFA for CCO. (A) A CCO problem consists of a set of discrete decision variables x jointly determining a global objective function $f\left(x\right)$ while simultaneously subjecting to multiple constraints $\left\{g_{k}\left(x\right)\right\}$. (B) Transformation of the CCO problem into ground state search of Ising model, seeking the spin configurations σ that minimize the Ising Hamiltonian. Parameters from the original problem map to coupling coefficients $J_{\mathrm{ij}}$ and external field terms $h_{i}$. (C) QIFA’s workflow: QIFA operates through an iterative loop consisting of 4 conceptual stages. Starting from an initialization of superposition state variables, the system undergoes gradient-based evolution driven by a time-dependent cost function. The resulting state is then projected onto a feasible discrete configuration, and the historically best solution is updated accordingly. A feedback mechanism guides subsequent evolution toward high-quality solutions, forming a closed-loop process that repeats until the time parameter reaches 𝑡 = 1, at which point the output solution is returned.

Each constraint acts only on a subset of the decision variables, which gives rise to overlapping and irregular feasible regions in the combinatorial configuration space. The coexistence of a global objective and multiple interacting constraints results in a feasible set that is highly structured and fragmented, constituting the intrinsic difficulty of CCO problems. This framework is schematically illustrated in Fig. 1A. The discrete decision variables are depicted as blue circular nodes, each representing a discrete variable $x_{1},\ldots ,x_{n}$​. These variables jointly determine the value of the global objective function, shown as a light-colored central region labeled $\min_{x}f\left(x\right)$, which represents the shared optimization goal. The effects of the constraints are illustrated by purple shaded regions that surround and restrict different subsets of variables.

It has been proven that the CCO problems can be mapped to the ground state search of Ising models [36]. As illustrated in ​​Fig. 1B​​, the discrete decision variables are encoded by spin variables $\sigma_{i}$ whose orientations correspond to candidate configurations $x_{i}$ of the original problem. The objective function together with constraint penalties are embedded into the energy landscape through coupling coefficients $J_{\mathrm{ij}}$ and local-field term $h_{i}$, yielding the system Hamiltonian with the following form:

$$H_{\text{ising}}=\sum_{\mathrm{ij}}^{n}J_{\mathrm{ij}}\sigma_{i}\sigma_{j}+\sum_{i}^{n}h_{i}\sigma_{i}$$ (1)

where the ground state of the Hamiltonian corresponds to the optimal feasible solution.

Traditional classical approaches for solving Ising-type optimization problems, such as SA, rely on sequential updates of spin configurations to explore the energy landscape through stochastic perturbations. As the system size increases, the search space grows rapidly, making it increasingly difficult to efficiently identify the global minimum. In contrast, quantum annealing describes the system state as evolving in a global Hilbert space, where a superposition state encodes multiple candidate configurations simultaneously. Beyond this parallel representation, quantum annealing is governed by an adiabatic process in which the system follows the ground state of a time-dependent Hamiltonian, enabling a continuous dynamical exploration of the energy landscape. The potential computational advantage of quantum annealing is often attributed to the combination of superposition-based representation and Hamiltonian-driven evolution, which together provide a search mechanism distinct from stochastic local updates.

To harness the parallelism in practical computation, we construct the QIFA that emulates the dynamical characteristics of quantum adiabatic evolution within a classical framework. The complete workflow is illustrated in Fig. 1C​​. The algorithm begins by encoding the superposition states of spins in the Ising model through classical variables. In the QIFA framework, each spin in the Ising model is in a superposition of spin-up and spin-down states. We introduce an analog variable $\theta_{i}\in \left[-\frac{\pi }{2},\;\frac{\pi }{2}\right]$ to characterize the orientation of the *i*th spin. The superposition state of the *i*th spin is represented as vector $S_{\theta_{i}}$:

$$S_{\theta_{i}}={\left[\sin\left(\frac{\theta_{i}}{2}+\frac{\pi }{4}\right),\;\cos\left(\frac{\theta_{i}}{2}+\frac{\pi }{4}\right)\right]}^{T}$$ (2)

Specifically, ${\left[1,\;0\right]}^{T}$ denotes the spin-up state, while ${\left[0,\;1\right]}^{T}$ denotes the spin-down state. The vector $S_{\theta_{i}}$ ​​represents a rotation on the Bloch sphere parameterized by angle $\theta_{i}$, mapping the computational basis spin-up and spin-down states to a superposition state with amplitudes given by the sine and cosine terms. Note that the probabilities corresponding to the 2 basis states are the squared moduli of the sine and cosine terms, thereby inherently satisfying the normalization condition. In contrast, the classical continuous relaxation lacks a direct physical correspondence and, consequently, do not impose a normalization requirement [9].

The expectation values of the Pauli operators $\sigma_{i}^{z}$ and $\sigma_{i}^{x}$​, which represent the energy contributions of each spin along the *z* axis and *x* axis, respectively, are straightforwardly computed as quadratic forms in the vector $S_{\theta_{i}}$. Specifically, we have:

$$S_{\theta_{i}}^{T}\sigma_{i}^{z}S_{\theta_{i}}=\sin\theta_{i}$$ (3)

$$S_{\theta_{i}}^{T}\sigma_{i}^{x}S_{\theta_{i}}=\cos\theta_{i}$$ (4)

The forms of Eqs. (3) and (4) are consistent with the standard expressions for the expectation values of observables in quantum mechanics. These expressions provide a direct relationship between the spin state and its corresponding energy contribution, which are key to the Ising model’s energy landscape.

The complete system state is represented as a tensor product of individual spin states, as follows:

$$S_{\theta }=S_{\theta_{1}}⊗S_{\theta_{2}}⊗\ldots ⊗S_{\theta_{n}}$$ (5)

Here, the vector $\theta ={\left[\theta_{1},\;\theta_{2},\;\ldots ,\;\theta_{n}\right]}^{T}$ collectively defines the configuration of all spins in the system, with each $\theta_{i}$ representing the orientation of the *i*th spin. As described in Eq. (2), each $S_{\theta_{i}}$ resides in a 2-dimensional Hilbert space. In Eq. (5), the full system state is constructed by taking the tensor product of *n* such 2-dimensional spin states, resulting in a composite Hilbert space of dimension $2^{n}$. This collective representation forms the complete system state. In essence, QIFA leverages this high-dimensional Hilbert space to explore multiple potential solutions simultaneously, enabling efficient global search over the solution space.

Afterward, evaluating the state described in Eq. (5), the iterative process is inspired by the quantum adiabatic theorem. The theorem states that if a quantum system starts in the ground state of a simple initial Hamiltonian, and this Hamiltonian is varied sufficiently slowly​ into a complex final Hamiltonian that encodes the target problem, the system will remain in the instantaneous ground state throughout the evolution, thereby arriving at the optimal solution. In the QIFA framework, this inspires the time-dependent Hamiltonian:

$$H\left(t\right)=\mathrm{t\gamma}H_{z}-\left(1-t\right)H_{x}$$ (6)

where $H_{z}$​ denotes the ​​matrix representation of the target Hamiltonian​​, $H_{x}$ represents the ​​matrix form of the transverse-field Hamiltonian​​, and γ serves as the ​​relative strength governing the dominance of $H_{z}$​ in the time-dependent system evolution.

$$H_{z}=\sum_{\mathrm{ij}}^{n}J_{\mathrm{ij}}\sigma_{i}^{z}\sigma_{j}^{z}+\sum_{i}^{n}h_{i}\sigma_{i}^{z}$$ (7)

and

$$H_{x}=\sum_{i}^{n}\sigma_{i}^{x}$$ (8)

It can be seen that at *t* = 0, Eq. (6) yields $\;H\left(0\right)=H_{x}$​, whose ground state corresponds to $S_{\left(\theta =0\right)}$ as described in Eq. (5), with all $\theta_{i}$ set to zero.

In contrast to quantum annealing, which follows unitary evolution under the Schrödinger equation, this framework simulates the system’s progression toward the ground state by updating variable θ through gradient descent. The time-dependent cost function is defined as the expectation value of the system’s Hamiltonian $H\left(t\right)$ at time *t*, which corresponds to the quadratic form of $H\left(t\right)$ under the vector $S_{\theta }$:

$$\begin{array}{c} C\left(t,\;\theta \right)=S_{\theta }^{T}H\left(t\right)S_{\theta }\\ =S_{\theta }^{T}\left(\mathrm{t\gamma}H_{z}-\left(1-t\right)H_{x}\right)S_{\theta }\\ =\mathrm{t\gamma}\left(z^{T}Jz+z^{T}\cdot h\;\right)-\left(1-t\right)x^{T}\cdot 1 \end{array}$$ (9)

where $\begin{array}{c} z={\left[\sin\theta_{1},\;\sin\theta_{2},\;\ldots \sin\theta_{n}\right]}^{T} \end{array}$ and $\begin{array}{c} x={\left[\cos\theta_{1},\;\cos\theta_{2},\;\ldots \cos\theta_{n}\right]}^{T} \end{array}$. The gradient of the cost function with respect to analog variables θ is given by:

$$\nabla_{\theta }C\left(t,\;\theta \right)=\mathrm{t\gamma}\left(2Jz+h\;\right)\circ x+\left(1-t\right)z$$ (10)

where ∘ denotes ​​element-wise multiplication​​ between vectors.

We update the analog variables θ via gradient descent (corresponding to the gradient descent module in Fig. 1C):

$$\theta_{t}=\theta_{t-\tau }-\eta \nabla_{\theta }C\left(t,\;\theta_{t}\right)$$ (11)

where $\theta_{t-\tau }$ is the analog variables obtained at the previous time, τ is the time evolution step size, and η is the step size in gradient descent. By minimizing $C\left(t,\;\theta \right)$, this gradient descent process iteratively adjusts θ to drive the vector $S_{\theta }$ toward the ground state of *H*(*t*) approximately at each step, thereby implementing an approximation of tracking the ground state described by the quantum adiabatic theorem.

At each iteration, a probabilistic projection step is applied to the current vector $\theta_{t}\;$to generate a discrete spin configuration. This projection is implemented through a roulette-wheel sampling strategy, in which spin orientations are selected according to probabilities induced by $\theta_{t}$​, producing a candidate solution $p_{t}$ that satisfies the problem constraints. The corresponding Hamiltonian value is evaluated for $p_{t}$, and the best feasible configuration encountered so far is recorded as $p_{\text{best}}$​ (corresponding to the project-update module in Fig. 1C). This roulette-wheel sampling is analogous to quantum measurement in quantum systems, where the measurement outcome is determined through probabilistic sampling based on the system’s quantum state.

In CCO problems, the energy landscape shaped by constraints is typically highly rugged, with feasible regions forming isolated basins separated by high-energy barriers. This rugged energy landscape easily traps searches in local minima. To escape these local traps and enhance search efficiency nearby the current optimal solution, we apply feedback adjustment to update variables $\theta_{t}$ in the direction of the optimal solution (corresponding to the feedback module in Fig. 1C):

$$\theta_{t}\leftarrow \left(1-\varsigma \right)\theta_{t}+\varsigma p_{\text{best}}$$ (12)

where *ς* is the feedback coefficient controlling the strength of this adjustment. This operation acts as a mechanism that integrates discrete optimization feedback into the dynamical evolution.

After completing the feedback process at time t, the time parameter is updated to t+τ. Subsequently, the time-dependent Hamiltonian evolves to $H\left(t+\tau \right)$, followed by the executions of gradient descent and project–feedback strategy to analog variables $\theta_{t+\tau }$. This iterative cycle repeats until the time parameter reaches t=1, concluding with the historical optimal solution $p_{\text{best}}$ as the final output. Through this evolution and feedback, QIFA provides a structured search process that promotes efficient convergence to high-quality feasible solutions.

### Numerical results

We take TSP as the benchmark to demonstrate the rapid solving capability of QIFA. TSP is a renowned CCO problem: Given a set of cities and pairwise distances, it seeks the shortest tour visiting each city exactly once and returning to the origin. This problem holds substantial practical relevance across domains including logistics, urban planning, bioinformatics, and chip design. The formal mathematical formulation of TSP, along with its corresponding Ising model Hamiltonian and constrained projection strategies, is detailed in the Methods section.

**Fig. 2.**
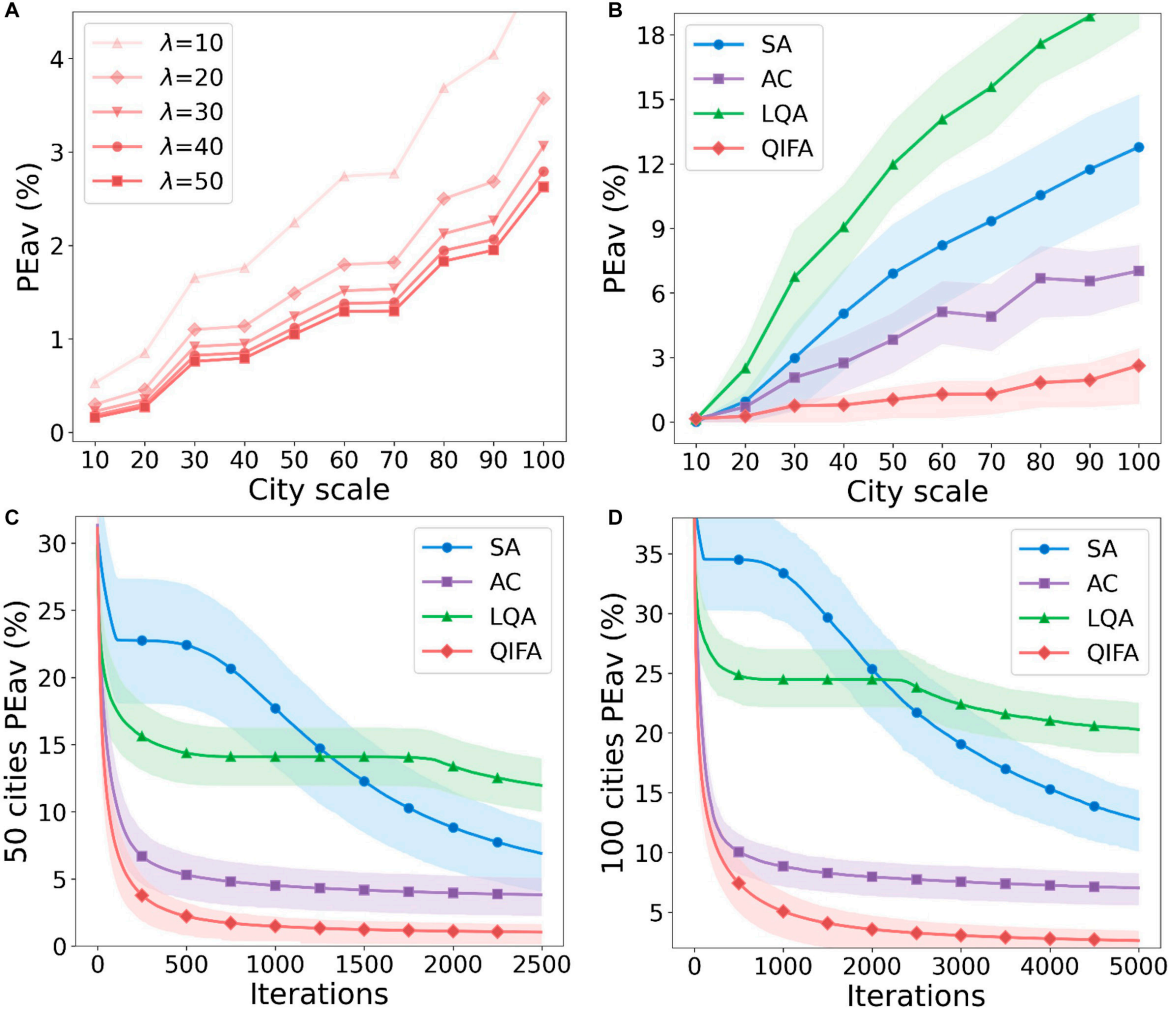
Numerical results on TSP instances across scales. (A) Solution quality of QIFA under varying iterative ratios λ for city scales from 10 to 100. (B) Solution quality comparison of SA, AC, LQA, and QIFA at city scales from 10 to 100, with iteration counts fixed at 50 times the city scale; lines show PEav from 100 repeated trials across 50 random instances, while shaded areas indicate interquartile ranges of percentage errors. (C) Solution quality versus iteration counts for SA, AC, LQA, and QIFA at 50-city scale, with lines representing PEav from 100 trials over 50 random maps and shaded areas depicting interquartile ranges of percentage errors. (D) Solution quality versus iteration counts at 100-city scale.

For city scales ranging from 10 to 100 in increments of 10, we generate​​ random city coordinates with the uniform distribution, producing 50 distinct TSP instances​​ per scale. Firstly, we examine the convergence properties of the QIFA by plotting its average percentage error [$\text{PEav}=\mathrm{avg}\left(\frac{E-E_{0}}{E_{0}}\times 100\%\right)$, with $E_{0}$ denoting the optimal solution and PEav quantifying solution quality, where lower values indicate superior performance] under varying iterative ratios λ (defined as λ=L/N, with L being the iteration count and N the city scale). As shown in Fig. 2A, negligible differences between λ=40 and λ=50 across all scales indicate that QIFA reaches stable convergence within this range.

We further compare QIFA with SA [3], AC [4], and local quantum annealing (LQA) [35]. Figure 2B illustrates the solution quality across scales, with all curves generated under iterative ratios λ=50. The red line depicts PEav trend of QIFA, while blue, purple, and green lines represent results of SA, AC, and LQA, respectively. The shaded areas indicate the interquartile range (25% to 75%) of percentage errors for each algorithm. All the results are generated from 100 repeated trials across 50 instances. QIFA consistently achieves lower percentage errors than competing algorithms, with performance advantages becoming more pronounced as the problem scale increases.

Figure 2C and D​​ details the convergence trends of the algorithms at scales of 50- and 100-city, respectively. The results for the remaining city scales are illustrated in Supplementary Information S1 The lines represent the PEav of different approach, while the shaded areas indicate the interquartile range of percentage errors. QIFA rapidly reduces PEav in early iterations and maintains consistently best solution quality compared with other algorithms as iteration count increases, demonstrating substantial advantages through faster convergence rates and superior solution accuracy.

To further quantify scalability, we analyze the number of iterations required to reach near-optimal solutions as a function of city size. As shown in Supplementary Information S2, QIFA empirically exhibits an approximately linear relationship between iteration count and problem scale within the randomly generated TSP instances considered here (city sizes from 10 to 100 with the uniform distribution of city coordinates). In contrast, SA shows a quadratic growth trend under the same solution accuracy, indicating a progressively increasing performance gap as the problem scale increases.

This near-linear scaling behavior can be interpreted through the dynamical structure of QIFA, which integrates 3 key quantum-inspired mechanisms: superposition encoding, adiabatic evolution, and projection–feedback. First, the superposition encoding represents the system state as a product-state parameterization in an exponentially dimensional Hilbert space. This encoding maintains a probabilistic description of multiple candidate configurations, allowing the algorithm to simultaneously explore the combinatorial landscape instead of sequentially evaluating individual states. This global representation substantially enhances the efficiency of the search process. Second, the optimization trajectory is guided by the adiabatic evolution of the system. The time-dependent Hamiltonian evolves gradually from an initial state that explores the entire solution space to the problem-specific Hamiltonian. This mirrors quantum adiabatic evolution, where the system follows the ground state of the time-dependent Hamiltonian, ensuring a smooth transition from exploration to exploitation of the problem landscape. Third, the projection-feedback mechanism plays a role analogous to quantum measurement, converting the continuous system state into feasible discrete configurations through probabilistic sampling. The feedback mechanism continuously updates the solution and adjusts the search direction, ensuring that the optimization remains within feasible regions of the parameter space while improving solution quality. Through the combined effect of these 3 mechanisms, the optimization trajectory evolves smoothly toward low-energy feasible states, avoiding the need for exhaustive enumeration of configurations.

Parameter sensitivity remains a critical challenge in heuristic optimization, often requiring extensive tuning to achieve peak performance and posing a major obstacle to real-world applicability. ​​To systematically evaluate this aspect,​​ we evaluate QIFA on a 20-city TSP benchmark across a wide parameter space, including Hamiltonian relative strength $\gamma \in \left[0.1,100\right]$, gradient descent step size $\eta \in \left[0.1,10\right]$​​, and feedback coefficient $\varsigma \in \left[0.1,0.99\right]$.

As detailed in Supplementary Information S3, QIFA consistently achieves low error rates (PEav < 0.8%) across all parameter combinations, indicating strong robustness to parameter variations. This robustness can be understood from the roles of the parameters in the dynamical framework. The parameter γ controls the relative dominance of the target Hamiltonian during evolution, influencing the balance between exploration and convergence speed. The step size η governs the magnitude of gradient-driven updates, primarily affecting convergence stability rather than final solution quality within the tested range. The feedback coefficient ς determines the strength of exploitation toward the best-known configuration, modulating the trade-off between global exploration and local refinement. Together, these parameters influence the convergence dynamics but do not fundamentally alter the structure of the optimization trajectory, which explains the observed robustness of QIFA across a broad parameter regime.

To further validate algorithmic availability, we benchmark QIFA against 20 standard instances from the ​​TSPLIB [37]​—a widely adopted dataset for TSP with known optimal solutions. ​​The selected instances are chosen to represent diverse problem characteristics varying city scale from 16 to 105 with different topological structures (Euclidean, geographic, and artificial configurations). As summarized in Supplementary Information S4, QIFA consistently achieves lower percentage errors than the comparison algorithms across the benchmark suite, with performance advantages becoming more pronounced for larger problem sizes. These results indicate that the efficiency and solution quality improvements observed in randomly generated instances extend to widely adopted real-world benchmarks.

To complement the solution quality analysis with computational efficiency, we further evaluate the runtime performance of QIFA and compare it with the Lin–Kernighan–Helsgaun (LKH) heuristic, a highly optimized solver widely used for TSP. All experiments are conducted on a CPU platform (Intel Core i7-9700, 3.00 GHz). To ensure a fair benchmarking setting, QIFA is executed up to its empirical saturation threshold of λ = 50. The LKH baseline is implemented using the standard Python wrapper (elkai.solve_int_matrix) with default parameters and without modification. The runtime for both methods is measured from the completion of the distance-matrix construction until the final solution is returned. Runtime statistics are obtained by averaging over 100 independent evaluations. Detailed runtime definitions are provided in Supplementary Information S5. QIFA achieves runtimes on the order of $10^{-1}\;\text{to}\;10^{0}$ s across the tested TSPLIB benchmark instances ranging from 29 to 100 cities, and operates within the same order of magnitude as LKH. Overall, these results demonstrate that QIFA attains high-quality solutions while maintaining high computational efficiency, supporting its practical applicability for combinatorial optimization problems.

The results presented above are simulated using classical computers. When implementing the QIFA on classical computers, spin superposition states require encoding through extensive binary expansions using high and low voltage levels, which fails to ​​substantially embody the properties of quantum superposition. On the other hand, recent investigations have shown that classical electric circuits can be used to simulate various topological physics [38–52], Schrödinger equation [53,54], and quantum algorithms [55], based on the similarity between circuit Laplacian and lattice Hamiltonian. In the following, we explore how to design circuit networks to perform the QIFA, which makes them have processing functions similar to that of quantum computation.

### Circuit theory for QIFA and simulation results

**Fig. 3.**
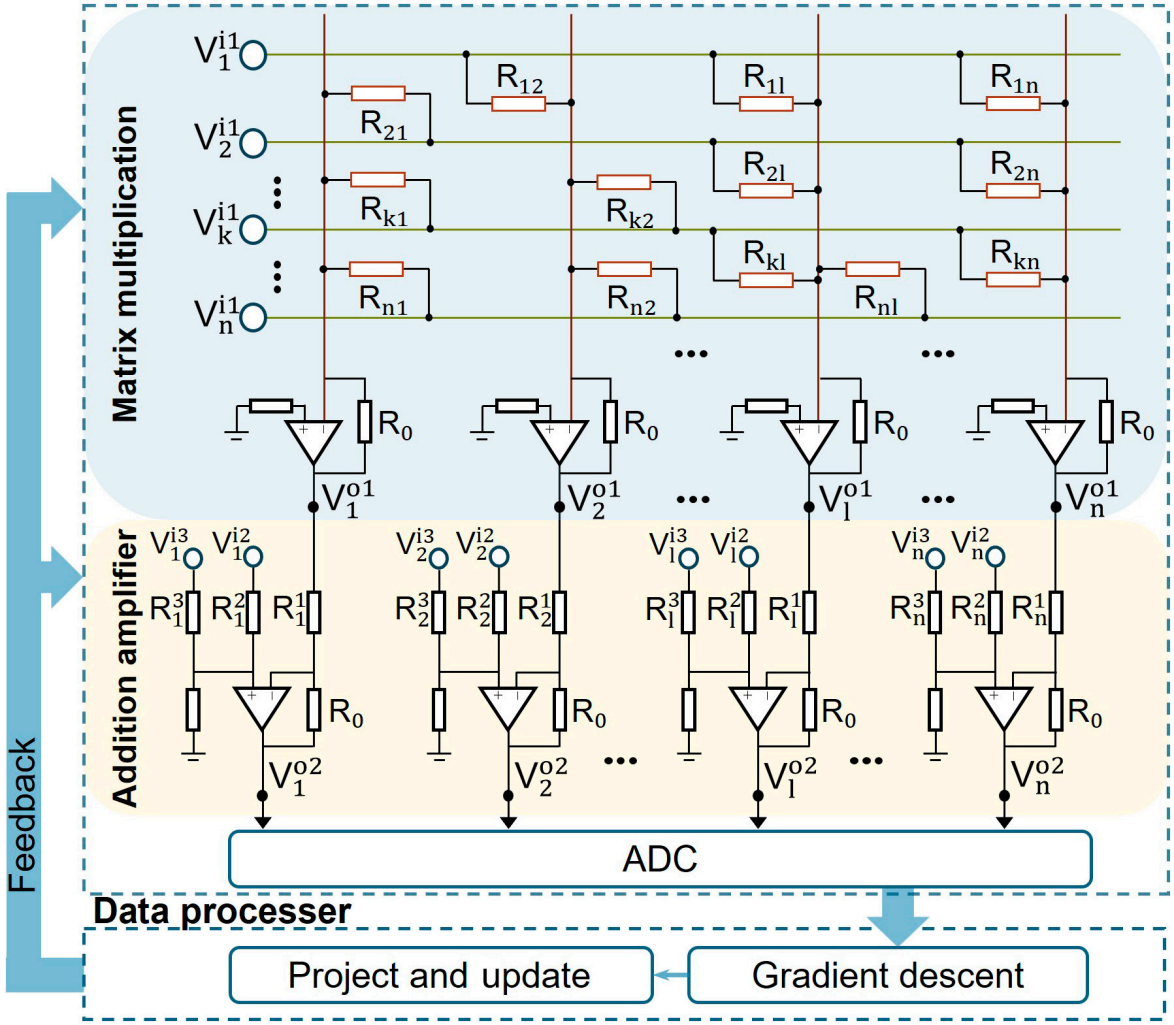
Schematic diagram of the classical circuit system. Blue: Matrix multiplication module. Yellow: Addition amplification module. The superposition state of spins is physically realized through voltage superposition across the nodes, and the coefficients of the Hamiltonian are modulated by resistors and input voltages. The data processor controls input to the classical circuit, receives ADC outputs as gradients of the cost function, and executes the gradient descent, constrained projection, and feedback adjustment.

The designed classical circuit to implement the full demonstration of QIFA is shown in Fig. 3, which consists of 2 modules, the matrix multiplication module in the blue area and the addition amplifier module in the yellow area.

The matrix multiplication module performs the core matrix–vector multiplication operation, which consists of n input nodes (indexed as k=1,2,…n) and n output nodes (indexed as l=1,2,…n). Each input node k connects to an external power signal $V_{k}^{i1}$, and resistor $R_{\mathrm{kl}}$ bridges every input node k and output node l. $R_{0}$ is the fixed resistance. Considering the virtual-short characteristics of operational amplifiers and applying Kirchhoff’s law, the voltage $V_{l}^{o1}$ ​at output node l satisfies:

$$V_{l}^{o1}=-\sum_{k}\frac{R_{0}V_{k}^{i1}}{R_{\mathrm{kl}}}$$ (13)

The addition amplifier module performs the weighted sum operation, which comprises n node. For each node l, an operational amplifier is connected to corresponding output $V_{l}^{o1}$ and external power signals $V_{l}^{i2}$ and $V_{l}^{i3}$​, incorporating with resistors $R_{l}^{1},\;R_{l}^{2}$, and $\;R_{l}^{3}$, respectively. Then, the output voltage $V_{l}^{o2}$ satisfies:

$$V_{l}^{o2}=R_{0}\left(\frac{V_{l}^{i2}}{R_{l}^{2}}+\frac{V_{l}^{i3}}{R_{l}^{3}}-\frac{V_{l}^{o1}}{R_{l}^{1}}\right)$$ (14)

By combining the voltages at all nodes, we can define the voltage sequence $V={\left[V_{1},\;V_{2},\;\ldots ,\;V_{n}\right]}^{T}$, manifesting as a superposition formulation of the voltages. In the matrix multiplication module, the input voltages $V^{i1}={\left[V_{1}^{i1},\;V_{2}^{i1},\;\ldots ,\;V_{n}^{i1}\right]}^{T}$ correspond to the superposition state of spins as shown in Eq. (3), which is physically realized as $V^{i1}=\text{sin}\;\theta$. Meanwhile, the second-order spin correlation coefficients $J_{\mathrm{kl}}$ of the Ising model are implemented by modulating resistors $R_{\mathrm{kl}}=\frac{R_{0}}{J_{\mathrm{kl}}}.$ After the first operational amplifier stage, according to Eq. (13), the output voltage sequence $V^{o1}={\left[V_{1}^{o1},\;V_{2}^{o1},\;\ldots ,\;V_{n}^{o1}\right]}^{T}$ is:

$$V^{o1}=-J\text{sin}\;\theta$$ (15)

In the addition amplifier module, the physical encoding of the transverse-field terms $h_{l}$ of the Ising model is achieved through the input voltages as $V_{l}^{i2}=h_{l}$ and the superposition state is realized as $V_{l}^{i3}=\text{sin}\;\theta_{l}$, respectively. The resistors are modulated as $R_{l}^{1}=\frac{R_{0}}{2\mathrm{t\gamma}\text{cos}\;\theta_{l}}$, $R_{l}^{2}=\frac{R_{0}}{\mathrm{t\gamma}\text{cos}\;\theta_{l}}$, and $R_{l}^{3}=\frac{R_{0}}{\left(1-t\right)}$ to scale the coefficients of the Hamiltonian. After the second operational amplifier stage, according to Eq. (14), the final output voltage sequence $V^{o2}={\left[V_{1}^{o2},\;V_{2}^{o2},\;\ldots ,\;V_{n}^{o2}\right]}^{T}$ is obtained as:

$$\begin{array}{c} V^{o2}=\mathrm{t\gamma}\left(2J\text{sin}\;\theta +h\;\right)\circ \text{cos}\;\theta +\left(1-t\right)\text{sin}\;\theta \end{array}$$ (16)

This resulting voltage output matches the gradient $\nabla_{\theta }C\left(t,\;\theta \right)$ in Eq. (10). After recording the output via analog-to-digital converter (ADC), the data processor executes gradient descent and constrained projection to generate trial solutions and update the historical optimal solution. Subsequently, the processor feeds the updated parameters back to the circuit for the next iteration cycle. This process iterates until the target iteration count is reached.

To validate the theoretical analysis, we perform numerical simulations (using LTspice software) of the circuit implementations for the TSP problems at varying scales, with specific circuit parameters detailed in the Methods section. For example, ​​we conduct comparative evaluations on TSPLIB instances including​​ the 29-city instance (bayg29), 48-city instance (att48), 76-city instance (eil76), and 100-city instance (rd100). Detailed solution progression is provided in ​​Supplementary Information S6​​. The simulation results consistently align with theoretical predictions, exhibiting matching convergence trends in solution energy as iterations increase.

### Experimental results

**Fig. 4.**
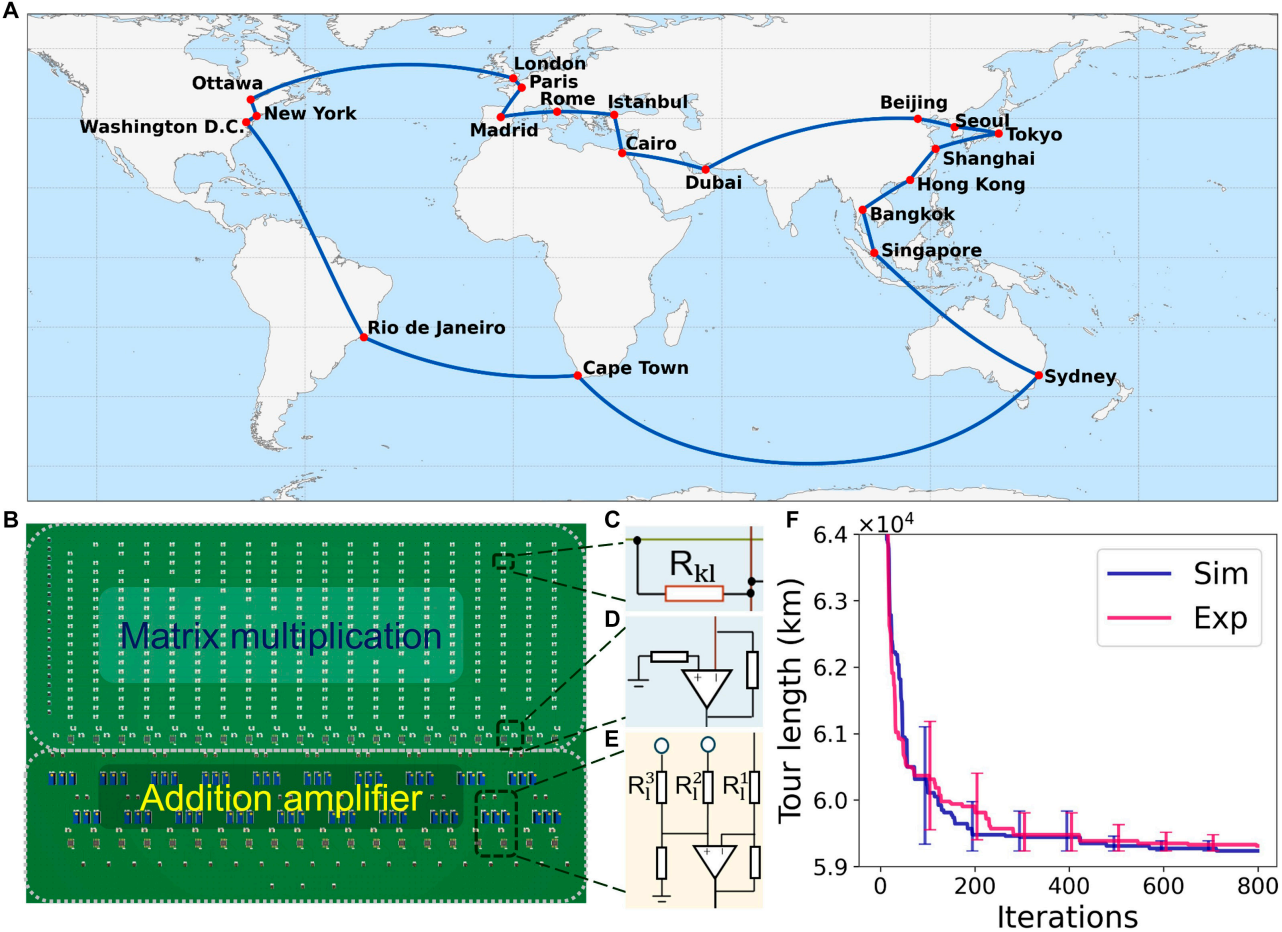
Experimental validation on 20-city global TSP. (A) Geographical distribution of 20 global cities and the optimal tour (blue lines), with coordinates mapped based on the WGS-84 ellipsoidal Earth model. (B) Layout of PCB hardware: Upper silkscreen area corresponds to the matrix multiplication module; lower section corresponds to the addition amplification module. (C) One precision chip resistor in the network of the matrix multiplication circuit module. (D) One block of the first-stage operational amplifier circuits converts the output currents of matrix multiplication circuit to voltages. (E) One block of the second-stage operational amplifier circuits in the addition amplification circuit module. (F) Simulated and experimental tour lengths versus iterations for QIFA on the 20-city global TSP, with the error bars indicating the fluctuation ranges.

To validate the practical problem-solving capability of executing QIFA on classical circuit systems, we select 20 major global cities as a case study for TSP, seeking the shortest tour traversing all cities. The names, locations, and optimal tour for these 20 cities are visualized in ​​Fig. 4A​. The specific latitude and longitude coordinates for each city are provided in​ Supplementary Information S7​​.

The designed circuit is fabricated on the printed circuit board (PCB) for solving the 20-city global TSP, as shown in ​​Fig. 4B. The upper region features the matrix multiplication module implemented via a 20 × 20 precision chip resistor network (Fig. 4C), ​​where the resistance values are configured to be inversely proportional to the spin correlation coefficients of the Ising model, thereby enforcing the TSP constraint that each city is visited exactly once.​ Subsequently, the first-stage operational amplifier circuit (​​Fig. 4D​​) converts the output current of the matrix multiplication module into the input voltage $V_{k}^{o1}$ for the next layer. The lower region constructs the addition amplifier module using 20 operational amplifiers and adjustable resistors, ​​where interconnecting resistance values represent intercity distances in the TSP problem​​. The second-stage operational amplifier circuit (​​Fig. 4E) performs addition and amplification to generate the output voltage $V_{k}^{o2}$ corresponding to the gradient. In the current prototype, parameter updates are performed in a hardware-in-the-loop manner: Circuit outputs are measured at each iteration, and the updated parameters are computed externally and manually tuned into the circuit, enabling iterative execution of the QIFA dynamics. Further details of the board configuration and experimental setup are provided in the Methods section.

​​Figure 4F​​ compares simulation and experimental result of tour lengths versus iteration counts for QIFA on the 20-city global TSP. The red line corresponds to the average of experimental results, and the blue line corresponds to the average of simulation results, with the error bars indicating the fluctuation ranges. We choose an appropriate density of the points to plot error bars per 100 iterations, which can not only keep the continuity of the results but also make the results visible and accurate. As iterations increase, both simulated and experimental results gradually decrease their fluctuation ranges, exhibiting consistent convergence trends approaching near-optimal tour solutions, and they together prove the effectiveness of our design. We further evaluate the impact of component tolerance on solution quality in Supplementary Information S8. The resulting tour lengths consistently converge across all tolerance levels. Notably, under the 1% tolerance condition—corresponding to the precision grade adopted in our physical experiments—the performance nearly matches that of the ideal tolerance-free scenario, demonstrating that the algorithm remains robust under real-world hardware constraints.

The above experiments demonstrate the availability of implementing QIFA in classical circuits, where the input–output relationship derived using Kirchhoff’s law establishes precise correspondence with the gradient of QIFA’s cost function. This classical circuit architecture leverages mature electronics manufacturing and control technologies to manifest effects equivalent to quantum state superposition through classical voltage–current superposition. Owing to inherent characteristics of classical circuits, this approach offers notable advantages: It can run in a normal environment without special requirements and has good scalability and stability.

Although the current physical setup operates as a proof of concept, the underlying analog circuit architecture is inherently compatible with automated digital control logic for fully integrated scaling. To implement QIFA in practical hardware systems, the aforementioned circuit requires collaboration with a field-programmable gate array (FPGA) for parameter modulations. In typical PCB circuit systems, FPGA clock frequencies operate at ~10 MHz, with unit response times on the order of 1 μs. Each iteration for an *N*-city TSP problem necessitates 2*N* parameter modulation operations, and the convergence requires approximately 50*N* iterations—totaling $100N^{2}$ parameter modulation response times. Consequently, FPGA-driven parameter modulation in PCB systems yields computation times of approximately 40 ms for 20-city instances, 250 ms for 50-city instances, and 1 s for 100-city instances. In application-specific integrated circuit (ASIC) systems with clock frequencies reaching ~1 GHz, QIFA is estimated to achieve computation times of approximately 0.4 ms for 20-city instances, 2.5 ms for 50-city instances, and 10 ms for 100-city instances, which is 2 to 3 orders lower than running on Intel Core-i7 CPU with the main frequency of 3 GHz.

## Discussion

Several prior studies employing quantum or quantum-inspired approaches have also adopted TSP as a benchmark for validation, and their reported performance on different problem sizes is summarized in Table 1. For instance, quantum annealing implemented on the D-wave quantum annealer achieved a PEav of 2.70% for a 22-city instance, which increased markedly to 25.91% for a 38-city instance, indicating clear scalability challenges [29]. Hybrid quantum-classical evolutionary algorithms combined classical evolution with NISQ devices, reporting a PEav of 7.4% for a 15-city TSP [56]. The quantum approximate optimization algorithm (QAOA), a widely used variational quantum algorithm for combinatorial optimization, reported a PEav of approximately 10% for a 5-city TSP [20]. In addition, quantum machine learning approaches based on equivariant quantum circuits attained PEav values of around 5% and 13% for 10- and 20-city TSP instances, respectively [20]. Quantum-inspired parallel annealing, which is also motivated by adiabatic evolution but does not explicitly incorporate constraint handling, achieved a PEav​ of about 5% for a 9-city TSP [11]. In addition, deep learning-based methods are a popular and active research approach for solving TSP in recent years [57,58]. Such approaches typically require problem-specific training procedures and considerable training time before deployment. Their computational workflow differs fundamentally from the iterative optimization paradigm considered in this work.

**Table 1.** Comparison of QIFA with representative quantum and quantum-inspired algorithms for solving TSP. This table compares the performance of QIFA with other quantum-inspired and quantum algorithms across various TSP problem sizes. The PEav (%) values represent the average percentage error relative to the optimal solution, with lower values indicating better solution quality. The Platform column specifies the hardware or simulation environment used for each algorithm, and the Remarks column provides additional context, including distinguishing features, scalability, and constraint handling capabilities.

Method	Size	PEav (%)	Platform	Remarks
Quantum annealing [29]	22	2.7%	D-wave quantum annealer	Performance degrades with increasing cities
	38	26%		
Hybrid quantum-classical evolutionary algorithm [56]	15	7.4%	Cloud-based NISQ platforms (IBM Quantum, AWS Braket)	Hybrid quantum inspired evolutionary algorithm with NISQ devices
QAOA [20]	5	~10%	Numerical study	Variational quantum algorithm; limited by the number of qubits
Equivariant quantum machine learning [20]	10	~5%	Numerical study	Leveraging the symmetry properties; effective for small-scale problems
	20	~13%		
Quantum-inspired parallel annealing [11]	9	5%	Analog memristor crossbar	Inspired by quantum annealing; no explicit constraint handling
	17	~10%		
QIFA (this work)	20	0.2%	Circuit network	Quantum-inspired Hamiltonian dynamics and projection–feedback
	100	~3%	Numerical study	

Notably, these quantum and quantum-inspired approaches generally suffer from either limited scalability or insufficient constraint handling, which leads to a rapid degradation of solution quality as the problem size increases. In contrast, the proposed QIFA synergistically combines the global exploration capability inherent in quantum-inspired paradigms with a projection–feedback strategy. This synergy enforces strict constraint satisfaction while enabling efficient navigation of the solution space, resulting in substantially improved solution quality, with a PEav of 0.2% for 20-city instances in experiment result and a PEav of approximately 3% for 100-city instances in numerical study.

On the other hand, although this article primarily focuses on TSP as an example, the QIFA framework demonstrates high flexibility and can be adapted to a wide range of CCO problems. For instance, when addressing KP, it suffices to map the problem onto its corresponding Ising model Hamiltonian, construct a time-dependent cost function for gradient descent, and design constraint-specific projection strategies under capacity limitations, along with feedback adjustments. This approach also achieves high-quality solutions for KP, demonstrating QIFA’s effectiveness across various problem types. The detailed QIFA theory and results for KP are presented in Supplementary Information S9 and S10. This structured approach enables seamless deployment across diverse problem domains, supporting QIFA as a versatile and scalable solution for solving a wide array of constrained optimization problems, beyond just TSP.

In summary, this paper proposes QIFA that delivers an efficient framework for the CCO problems. The core innovation is encoding spin superposition states with classical variables, which enables gradient-driven global search with parallel capabilities. Meanwhile, project–feedback strategy accelerates convergence by intensifying exploration in high-quality regions. We demonstrate the feasibility of implementing the QIFA framework in classical circuit systems, whose architecture aligns with mainstream semiconductor manufacturing capabilities. This approach operates robustly in standard environments without quantum-specific conditions, exhibiting scalability and stability. Leveraging ASICs or FPGAs for parallel parameter modulation, QIFA holds promise for real-time optimization in large-scale practical applications, thereby facilitating the deployment of industrial-grade real-time optimization systems.

## Methods

### The QIFA theory for TSP

TSP can be formulated as a quadratic assignment problem by defining a set of binary variables representing whether each edge belongs to the solution tour. Specifically, for a graph *G* = (*V*, *E*), TSP is expressed as:

$$\text{min}\sum_{\left(i,\;j\right)\in E}d_{\mathrm{ij}}a_{\mathrm{ij}}$$ (17)

$$s.t.\sum_{i\in V\backslash \left\{j\right\}}a_{\mathrm{ij}}=1,j\in V$$ (18)

$$\sum_{j\in V\backslash \left\{i\right\}}a_{\mathrm{ij}}=1,i\in V$$ (19)

$$\sum_{i\in S}\sum_{j\notin S}a_{\mathrm{ij}}\geq 1,S⊊V,\left|S\right|\geq 2\;$$ (20)

Here, binary variables $a_{\mathrm{ij}}$ indicate whether edge $E_{\mathrm{ij}}$ belongs to the solution tour, and $d_{\mathrm{ij}}$ denotes the length of edge $E_{\mathrm{ij}}$. Equation (17) represents the optimization objective, corresponding to the total length of the solution tour; Eqs. (18) and (19) are equality constraints ensuring exactly one path departing from and terminating at each city, respectively; Eq. (20) is an inequality constraint guaranteeing that the solution contains exactly one ring.

In our algorithm, the inequality constraint [Eq. (20)] is implemented during the constrained projection via the roulette-wheel strategy, while the equality constraints [Eqs. (18) and (19)] are incorporated into the objective term:

$$\begin{array}{c} \begin{array}{c} H=\sum_{\left(i,\;j\right)\in E}d_{\mathrm{ij}}a_{\mathrm{ij}}+D\sum_{i}{\left(\sum_{j\in V\backslash \left\{i\right\}}a_{\mathrm{ij}}-1\right)}^{2}\\ +D\sum_{j}{\left(\sum_{i\in V\backslash \left\{j\right\}}a_{\mathrm{ij}}-1\right)}^{2} \end{array} \end{array}$$ (21)

where $D=\max\left\{d_{\mathrm{ij}}\right\}$. After variable substitution and rearrangement, Eq. (21) yields the Ising model formulation for TSP:

$$\begin{array}{c} H_{z}^{\mathrm{TSP}}=\sum_{\left(i,\;j\right),\left(k,\;l\right)\in E}J_{\mathrm{ij},\mathrm{kl}}\sigma_{\mathrm{ij}}^{z}\sigma_{\mathrm{kl}}^{z}+\sum_{\left(i,\;j\right)\in E}^{n}h_{\mathrm{ij}}\sigma_{\mathrm{ij}}^{z} \end{array}$$ (22)

where

Jij,kl=D4,i=korj=l,Jij,kl=0,otherwise. (23)

$$h_{\left(i,\;j\right)}=\frac{d_{\mathrm{ij}}}{2}-\left(n-1\right)D$$ (24)

In QIFA for TSP, the time-dependent Hamiltonian is

$$H^{\mathrm{TSP}}\left(t\right)=\mathrm{t\gamma}H_{z}^{\mathrm{TSP}}-\left(1-t\right)H_{x}^{\mathrm{TSP}}$$ (25)

We introduce analog variables $\begin{array}{c} \theta ={\left[\theta_{11},\;\theta_{12},\;\ldots ,\;\theta_{\mathrm{ij}},\;\ldots ,\;\theta_{\mathrm{NN}}\right]}^{T} \end{array}$, where $\theta_{\mathrm{ij}}\in \left[-\frac{\pi }{2},\;\frac{\pi }{2}\right]$ represents the orientation of the *ij*th spin. The time-dependent cost function corresponds to the quadratic form of $H^{\mathrm{TSP}}\left(t\right)$ with the vector $S_{\theta }$:

$$C\left(t,\;\theta \right)=S_{\theta }^{T}H^{\mathrm{TSP}}\left(t\right)S_{\theta }$$ (26)

The partial derivative of the cost function with respect to parameter $\theta_{\mathrm{ij}}$ is given by:

$$\begin{array}{c} \begin{array}{c} \frac{\partial C\left(t,\;\theta \right)}{\partial \theta_{\mathrm{ij}}}=\mathrm{t\gamma}\left(\frac{\lambda }{2}\sum_{l}z_{\mathrm{il}}+\frac{\lambda }{2}\sum_{k}z_{\mathrm{kj}}+\frac{d_{\mathrm{ij}}}{2}-\left(N-1\right)D\;\right)x_{\mathrm{ij}}+\left(1-t\right)z_{\mathrm{ij}} \end{array} \end{array}$$ (27)

To obtain constraint-satisfying trial solutions $p_{t}$ from θ, the constrained projection strategy is configured as follows: Let Y denote the set of visited city indices and i the index of the last visited city. The probability $P_{\mathrm{ij}}$ for edge $E_{\mathrm{ij}}$ to be chosen in the solution ​​tour​​ via roulette-wheel selection is set to:

$$P_{\mathrm{ij}}=\left\{\begin{array}{cc} \frac{{\left(1+z_{\mathrm{ij}}\right)}^{2}d_{\mathrm{ij}}^{\beta }}{\sum_{l\notin C}{\left(1+z_{\mathrm{il}}\right)}^{2}d_{\mathrm{il}}^{\beta }}, & j\notin Y\\ 0, & j\in Y \end{array}\right.$$ (28)

where $z_{\mathrm{ij}}=\sin\theta_{\mathrm{ij}}$ and β is the edge-length heuristic factor. Because the roulette-wheel selection is performed sequentially with the visited set updated after each selection, the procedure explicitly enforces connectivity and prevents the formation of subtours, guaranteeing that the trial solution $p_{t}$ contains ​​exactly one tour​​ with each city visited exactly once.

### The circuit parameters for TSP

For an N-city TSP problem mapped to an Ising model with $N^{2}$ spins, the standard QIFA implementation would require $N^{2}$ circuit nodes. However, leveraging the ​​sparsity and symmetry​​ revealed in Eq. (27), we reduce hardware complexity by utilizing N circuit nodes to compute the cost function gradient through 2N repeated computations per iteration. The experimental configuration operates as follows:

In the *i*th computation for component $\frac{D}{2}\mathrm{t\gamma}\sum_{l}z_{\mathrm{il}}x_{\mathrm{ij}}$: Resistors between input node k and output node lare modulated as $\begin{array}{c} R_{\mathrm{kl}}=\frac{4R_{0}}{D} \end{array}$. Resistors in node j are set to $\begin{array}{c} R_{j}^{1}=\frac{R_{0}}{2\mathrm{t\gamma}x_{j}},R_{j}^{2}=0,R_{j}^{3}=0 \end{array}$. Input voltages are set to $V_{j}^{i1}=z_{\mathrm{ij}},V_{j}^{i2}=0,V_{j}^{i3}=0$, yielding output voltage in *i*th computation $V_{i,j}^{o2}=\frac{\lambda }{2}\mathrm{t\gamma}\sum_{l}z_{\mathrm{il}}x_{\mathrm{ij}}$. In the subsequent $\left(n+j\right)$ th computation for component $\begin{array}{c} \left(\frac{\lambda }{2}\sum_{k}z_{\mathrm{kj}}+\frac{d_{\mathrm{ij}}}{2}-\left(N-1\right)D\right) \end{array}$$\begin{array}{c} x_{\mathrm{ij}}+\left(1-t\right)z_{\mathrm{ij}} \end{array}$, resistors in node i are set as $\begin{array}{c} R_{i}^{1}=\frac{R_{0}}{2\mathrm{t\gamma}x_{i}},R_{i}^{2}= \end{array}$$\begin{array}{c} \frac{R_{0}}{\mathrm{t\gamma}x_{i}},R_{i}^{3}=\frac{R_{0}}{\left(1-t\right)} \end{array}$. Input voltages are set to $\begin{array}{c} V_{i}^{i1}=z_{\mathrm{ij}},V_{i}^{i2}=\frac{d_{\mathrm{ij}}}{2}- \end{array}$$\begin{array}{c} \left(N-1\right)D,V_{i}^{i3}=z_{\mathrm{ij}} \end{array}$, producing output voltage $\begin{array}{c} V_{n+j,i}^{o2}=\mathrm{t\gamma} \end{array}$$\begin{array}{c} \left(\frac{\lambda }{2}\sum_{k}z_{\mathrm{kj}}+\frac{d_{\mathrm{ij}}}{2}-\left(N-1\right)D\;\right)x_{\mathrm{ij}}+\left(1-t\right)z_{\mathrm{ij}} \end{array}$. The combined output $\frac{\partial C\left(t,\;\theta \right)}{\partial \theta_{\mathrm{ij}}}$ = $V_{i,j}^{o2}+V_{n+j,i}^{o2}$ matches Eq. (27). These voltages are recorded via ADC, after which the processor executes gradient descent and project–feedback strategy.

### Details for experimental implementation

We utilize EasyEDA software to design the PCBs, which are fabricated at a local foundry. The precision chip resistors used in the circuit are manufactured by RESI Company, featuring a 0603 package with a tolerance of 1%. The precision operational amplifier (model AD8676ARZ-REEL7) operates at ±18V and features a low input bias current (Ib = 2 nA), a low input offset voltage (Vos = 12 μV), and a low input offset current (Ios = 1 nA), all of which have negligible impact on measurement results. We select a 1-kΩ grounding resistor for the non-inverting input terminal. The resistance values of all resistors connected between the inverting input terminal and the input voltage, as well as the feedback resistor $R_{0}$, are 1 kΩ. The potentiometer (model 3296W-1-103-8mm) provides an adjustable resistance range of 0 to 10 kΩ.

The system power supply is provided by multiple programmable DC voltage sources of model UDP3305E, which can output a DC voltage ranging from −30 V to +30 V with a resolution of 10 mV. The PCB is connected to the voltage source via a cable with 4-mm lantern plugs on one end and alligator clips on the other.

The measured quantities of our circuit are the output voltages corresponding to each set of input voltages. In our experiment, we use a multimeter (model VC9810A, 1-mV resolution) to collect the output voltage data.

## Data Availability

The data that underlie the plots within this paper and other findings of this study are available from the corresponding authors on reasonable request. A demonstration code of QIFA is available at the GitHub repository: https://github.com/crypsis98/QIFA. Additional codes related to this study are available from the corresponding author upon reasonable request.
