# Supplementary material for: Quantum-Inspired Fast Algorithm and Circuit Realization for Constrained Combinatorial Optimization Problem
Source: Research (Wash D C). 2026 Jun 29;9:1345. doi: 10.34133/research.1345 (PMC13311256; doi:10.34133/research.1345)
Supplement: Supplementary 1 — Supplementary Information S1 to S10 Figs. S1 to S6 Tables S1 and S2 [file research.1345.f1.pdf]

Supplementary Information for
Quantum-inspired fast algorithm and circuit realization for constrained combinatorial optimization problem

Haosen Chen*, Shailan Deng*, Tian Chen$ and Xiangdong Zhang+

Key Laboratory of advanced optoelectronic quantum architecture and measurements of Ministry of Education, School of Physics, Beijing Institute of Technology, 100081, Beijing, China

**These authors contributed equally to this work.* [+$]*Author to whom any correspondence should be addressed. E-mail:* zhangxd@bit.edu.cn, chentian@bit.edu.cn

Supplementary Information S1. Comparative convergence trends of solution quality across city scales.
Supplementary Information S2. Scaling trend for iterations-to-solution.
Supplementary Information S3. Parameter robustness of QIFA.
Supplementary Information S4. Verification on TSPLIB benchmark instances.
Supplementary Information S5: Runtime performance of QIFA for TSPLIB Benchmarks.
Supplementary Information S6. Simulation and comparative results for TSPLIB Benchmarks.
Supplementary Information S7. The latitude and longitude coordinates of the 20 major cities.
Supplementary Information S8. Disorder of the circuit components.
Supplementary Information S9. The QIFA theory for Knapsack Problem.
Supplementary Information S10. The QIFA result for KP.

Supplementary Information S1: Comparative convergence trends of solution quality across city scales.

Fig. 2c and 2d detail the convergence trends across algorithms at scales of 50 and 100 cities. Here, Fig. S1 presents the convergence trends of solution quality as iteration count increases at the remaining city scales respectively. The red/blue/purple/green lines represent the PEav of QIFA, SA, AC and LQA respectively, while the shaded areas indicate the interquartile range of percentage errors.

As shown in Fig. S1a, for the 10-city scenario, the solution space is relatively small, and the impact of local minima induced by constraint terms is negligible. Consequently, LQA, AC, and QIFA all rapidly approach near-optimal solutions with high convergence speeds, while SA also exhibits a relatively fast descending trend. However, as the city scale increases (20-city in Fig. S1b to 90-city in Fig. S1h), the problem complexity grows significantly. Although the results of LQA and AC still

show rapid downtrend in the early iterations, they become increasingly affected by local minima caused by constraints, resulting in solution quality progressively deviating from the optima as city scale growing up. SA , with lacking on global solution-space perception, relies solely on random exploration within the neighborhood of the current solution, leading to a notable decline in search efficiency as problem scale expands. In contrast, QIFA benefits from superposition encoding, which enhances global perception, enabling consistently fast convergence across city scales ranging from 10 to 100. Furthermore, by leveraging a projection–feedback strategy , QIFA improves search efficiency in high-quality

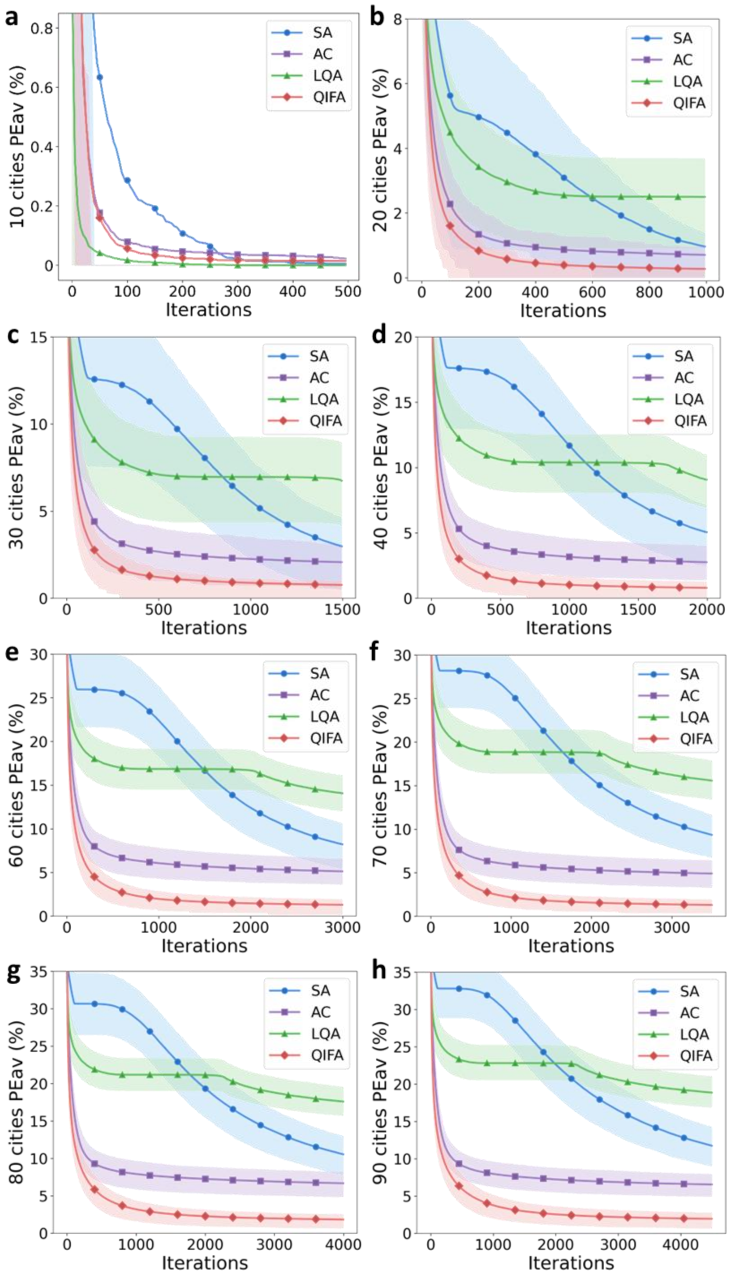

solution regions, effectively avoids local minima, and achieves superior solution quality.

Fig. S1. **Convergence trends across city scales.** Illustrate how solution quality grows as iteration count increases at different scales. **a**, 10 cities. **b,** 20 cities. **c,** 30 cities. **d,** 40 cities. **e,** 60 cities. **f,** 70 cities. **g,** 80 cities. **h,** 90 cities. The lines represent the PEav across algorithms and the shaded areas indicate the interquartile range of percentage errors.

Supplementary Information S2: Scaling trend for iterations-to-solution.

In our previous analysis, we compare the convergence trends of various algorithms across different city scales, demonstrating that QIFA achieves faster iteration speeds and higher solution quality compared to other methods. Here, we specifically examine the relationship between city scale (ranging from 10 to 100) and the number of iterations required to reach a target solution quality (iterations-to-solution) for both QIFA and SA. We numerically compute the number of iterations required for SA to converge at various city scales, while also recording the number of iterations needed for QIFA to achieve the same solution quality.

As illustrated in Fig. S2, the relationship between city scale (denoted as N) and the count of iterations (denoted as L) required to reach near-optimal solutions is compared for QIFA and SA. QIFA exhibits an approximately linear increase in iteration count as the city scale grows from 10 to 100 nodes (red diamond markers) fitted with red dashed line as L=10.7N, whereas SA shows a quadratic increase (blue circular markers) fitted with blue dashed line as L=2.57N^2. This indicates that for larger problem instances, the iteration count of QIFA grows at a significantly slower rate than that of SA. The linear scaling of QIFA can be attributed to its quantum-inspired continuous superposition encoding and projection–feedback strategy, which enhance global search capability and help avoid local optima. In contrast, the quadratic scaling of SA arises from its classical annealing mechanism, which becomes progressively inefficient as the problem size increases. This divergence in scaling behavior leads to a progressively widening performance gap as problem size increases, which clearly demonstrate the superior scalability of QIFA and its strong potential for real-world applications, overcoming limitations typical of classical algorithms such as SA in large-scale constrained optimization problems.

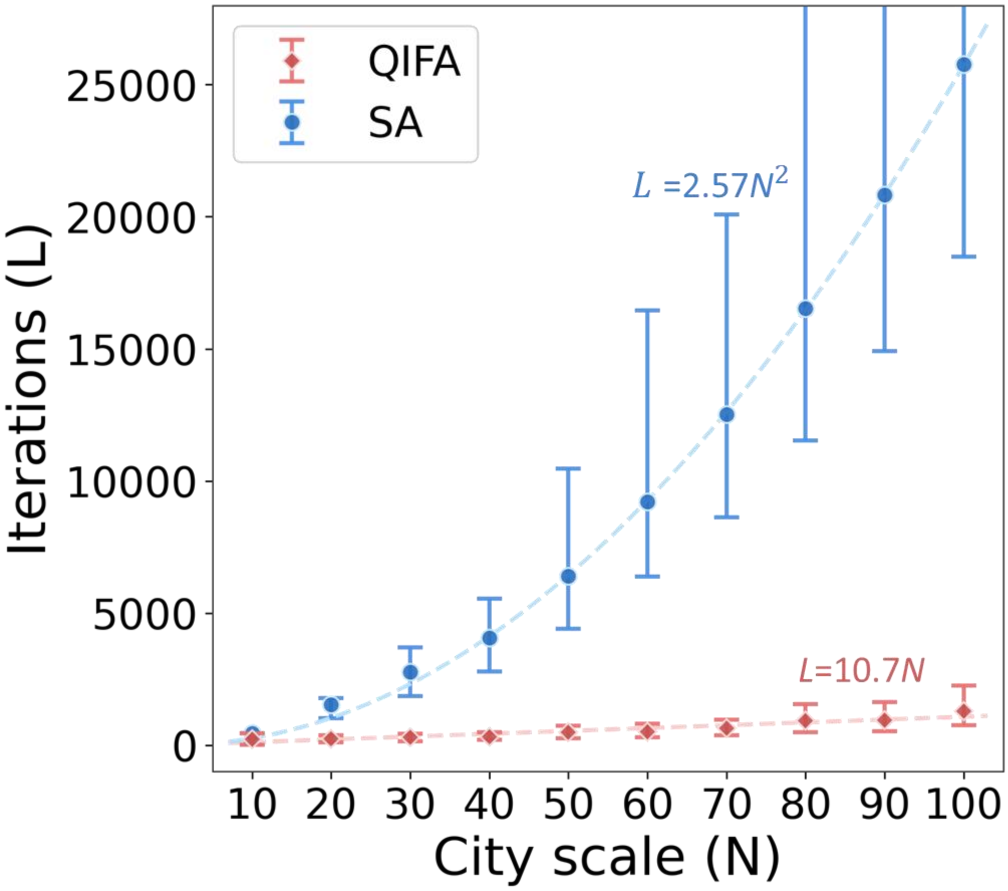

Fig. S2. Scaling trend for iterations-to-solution. Illustrate the scaling relationship between city scale and the number of iterations required to reach near-optimal solutions for QIFA and SA. The red diamond markers represent the number of iterations required by QIFA across different city scales (ranging from 10 to 100), with error bars indicating the interquartile ranges. The red dashed line corresponds to a first-order linear fit of QIFA's iteration count, demonstrating an approximately linear scaling with problem size. In contrast, SA—represented by blue circular markers—exhibits a quadratic increase in iterations to achieve comparable solution quality as QIFA as the city scale grows.

Supplementary Information S3: Parameter robustness of QIFA .

Parameter sensitivity remains a critical challenge in heuristic optimization, often requiring extensive tuning to achieve peak performance and posing a major obstacle to real-world applicability. This is particularly pronounced in algorithms relying on discrete or stochastic representations, where small parameter deviations can lead to drastically degraded solutions or complete convergence failure.

Here, we evaluate QIFA on 20-city TSP instance across 1000 repeated trials at each parameter combination, with: Hamiltonian relative strength $\gamma \in [0.1,100]$, gradient descent step size $\eta \in [0.1,10]$ and reward coefficient $\varsigma \in [0.1,0.99]$. The color-coded PEav (Fig. S3) consistently remains below 0.8% throughout the parameter space, demonstrating QIFA's robustness to parameter selection. This remarkable parameter insensitivity stems from QIFA's core design: the continuous spin representation creates smooth gradient landscapes that are less susceptible to parameter variations, while the projection-feedback strategy naturally compensates for suboptimal parameter choices by reinforcing constraint satisfaction. The consistent performance across these diverse parameter combinations suggests that QIFA requires minimal parameter tuning for practical deployment, significantly lowering the barrier for real-world applications.

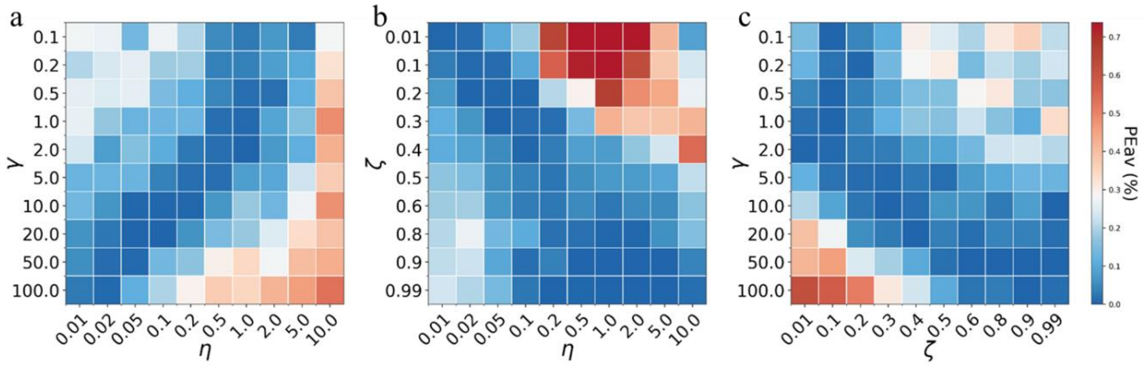

Fig. S3. PEav of QIFA under varied parameters. (a) Results for Hamiltonian relative strength γ vs. gradient descent step size η. (b) Results for reward coefficient ς vs. gradient descent step size η. (c) Results for Hamiltonian relative strength γ vs. reward coefficient ς.

Supplementary Information S4: Verification on TSPLIB benchmark instances.

For twenty TSPLIB benchmark instances with city scales ranging from 16 to 105, we compare the solution quality of the QIFA against SA, AC and LQA. Each algorithm executes 100 repeated trials per instance with a fixed total iteration counts of 5,000. Results demonstrate QIFA's clear superiority over SA, AC, and LQA in average solution quality across all twenty instances, with its advantage becoming increasingly pronounced as problem scale grows.

Ins	opt	SA		AC		LQA		**QIFA**	
		avg	PEav	avg	PEav	avg	PEav	**avg**	**PEav**
ulysses16	6859	6862.75	0.05	6884.61	0.37	6945.94	1.27	**6859.00**	**0.00**
ulysses22	7013	7051.05	0.54	7097.66	1.21	7260.28	3.53	**7015.36**	**0.03**
gr24	1272	1283.12	0.87	1277.08	0.40	1343.99	5.66	**1272.54**	**0.04**
fri26	937	949.75	1.36	937.04	0.00	957.34	2.17	**937.54**	**0.06**
bayg29	1610	1619.80	0.61	1663.11	3.30	1758.92	9.25	**1615.75**	**0.36**
bays29	2020	2030.02	0.50	2052.70	1.62	2169.85	7.42	**2028.53**	**0.42**
att48	10628	10897.92	2.54	11284.52	6.18	11820.81	11.22	**10696.80**	**0.65**
gr48	5046	5178.52	2.63	5384.44	6.71	5707.32	13.11	**5126.25**	**1.59**
eil51	426	440.62	3.43	451.40	5.96	491.99	15.49	**430.10**	**0.96**
berlin52	7542	7958.56	5.52	7706.34	2.18	8508.39	12.81	**7641.04**	**1.31**
st70	675	720.21	6.70	731.62	8.39	791.85	17.31	**693.00**	**2.67**
eil76	538	578.93	7.61	570.04	5.96	636.88	18.38	**546.99**	**1.67**
pr76	108159	116508.91	7.72	120782.46	11.67	128586.32	18.89	**113483.64**	**4.92**
gr96	55209	61075.83	10.63	59819.00	8.35	66071.06	19.67	**57375.92**	**3.92**
kroA100	21282	23894.71	12.28	23221.95	9.12	25816.89	21.31	**22011.86**	**3.43**
kroC100	20749	23547.67	13.49	21869.20	5.40	24902.02	20.02	**21122.67**	**1.80**
kroD100	21294	23870.57	12.10	23120.98	8.58	25474.97	19.63	**22017.78**	**3.40**
rd100	7910	8973.33	13.44	8603.66	8.77	9659.47	22.12	**8056.93**	**1.86**
eil101	629	707.49	12.48	706.18	12.27	784.12	24.66	**663.54**	**5.49**
lin105	14379	16417.40	14.18	15173.34	5.52	16952.23	17.90	**14704.72**	**2.27**

Table S1. **Comparative verification on TSPLIB benchmarks**. The column labels respectively denote: Ins, the instance name of the TSPLIB benchmark; opt, the optimal tour length; avg, the average tour length across 100 trials; PEav, the average percentage error of solution lengths relative to the optima.

Supplementary Information S5: Runtime performance of QIFA for TSPLIB Benchmarks.

To further evaluate the computational efficiency of QIFA, we measure its runtime performance on several standard TSP benchmarks from the TSPLIB library, including bayg29, att48, eil76, and rd100, containing 29, 48, 76, and 100 cities, respectively. The results are compared with the Lin–Kernighan–Helsgaun (LKH) heuristic, which is widely regarded as one of the most efficient classical solvers for TSP. All runtime experiments are conducted on a desktop workstation equipped with an Intel Core i7-9700 CPU (3.00 GHz) and 8 GB RAM. Both QIFA and LKH are executed on the same CPU platform under identical experimental conditions to ensure a fair comparison.

To ensure rigorous reproducibility, the execution times reported for both algorithms in Table S2 represent the average runtime evaluated over 100 independent evaluations for each TSPLIB instance. The timing for both methods uniformly commences immediately after the completion of the pairwise distance-matrix construction and concludes when the final output solution is successfully generated. Standard preprocessing steps, such as file standard loading and coordinate parsing, are excluded. Prior to formal timing measurements, a warm-up execution is performed for both QIFA and LKH to eliminate one-time initialization overheads associated with parallel computation resources and runtime environments, thereby ensuring stable and reproducible timing measurements.

For the LKH baseline, the standard Python implementation (elkai.solve_int_matrix) is used with its default settings and without parameter tuning. Each evaluation corresponds to one call to elkai.solve_int_matrix(), within which LKH performs its default multi-start search configuration with 10 parallel searches initialized from independent random seeds, and the best solution among them is returned. For QIFA, the recorded runtime comprehensively covers all core computational stages within the framework, explicitly including the Ising Hamiltonian construction, gradient descent, and the projection-feedback operations.

The runtime comparison results are summarized in Table S2. As expected, LKH achieves very fast runtimes due to its extensive algorithmic engineering and problem-specific heuristics. In contrast, QIFA is designed as a general-purpose dynamical optimization framework rather than a specialized TSP solver. Despite this difference in design objectives and implementation, the runtime of QIFA remains within the same order of magnitude as LKH across the tested

instances. For the att48 instance, QIFA exhibits slightly shorter runtime, which can be attributed to earlier convergence under the adopted stopping criterion, albeit with a relatively larger optimality gap.

Overall, these results indicate that QIFA achieves competitive computational efficiency while maintaining strong optimization performance.

Ins	opt	LKH			**QIFA**		
		avg	PEav (%)	Runtime (s)	avg	PEav (%)	**Runtime (s)**
bayg29	1610	1610	0	**0.071**	1628.58	0.36	**0.105**
att48	10628	10661	0.311	**0.325**	10732.37	0.98	**0.304**
eil76	538	538	0	**0.998**	547.27	1.72	**1.265**
rd100	7910	7922	0.15	**1.619**	8056.93	1.86	**2.547**

Table S2. Runtime comparison between QIFA and the LKH solver on TSPLIB benchmarks. For each instance, the optimal tour length (Opt), average solution value (avg), average percentage error (PEav), and runtime are reported. LKH results are obtained using the standard LKH solver, while QIFA results correspond to the proposed algorithm implemented on a CPU platform.

Supplementary Information S6: Simulation and comparative results for TSPLIB Benchmarks.

As shown in Fig. S4, we conduct comprehensive simulations on four representative TSPLIB benchmark instances—bayg29, att48, eil76, and rd100—to evaluate both circuit-simulated and theoretical performance of QIFA alongside comparative approach (SA, AC, and LQA). Across all instances, the circuit-simulated results of QIFA (orange curve) closely match its theoretical values (red curve), demonstrating high consistency between analog simulation and mathematical modeling. Furthermore, QIFA significantly outperforms all classical counterparts throughout the convergence process. In instance rd100 (Fig. S4d), which contains 100 cities, QIFA achieves a final tour length within 2.89% of the known optimum, whereas SA, AC, and LQA exhibit considerably higher errors and slower convergence rates. These results

highlight not only the precision of the proposed circuit implementation but also the scalability and practical effectiveness of QIFA across varying problem sizes and topologies.

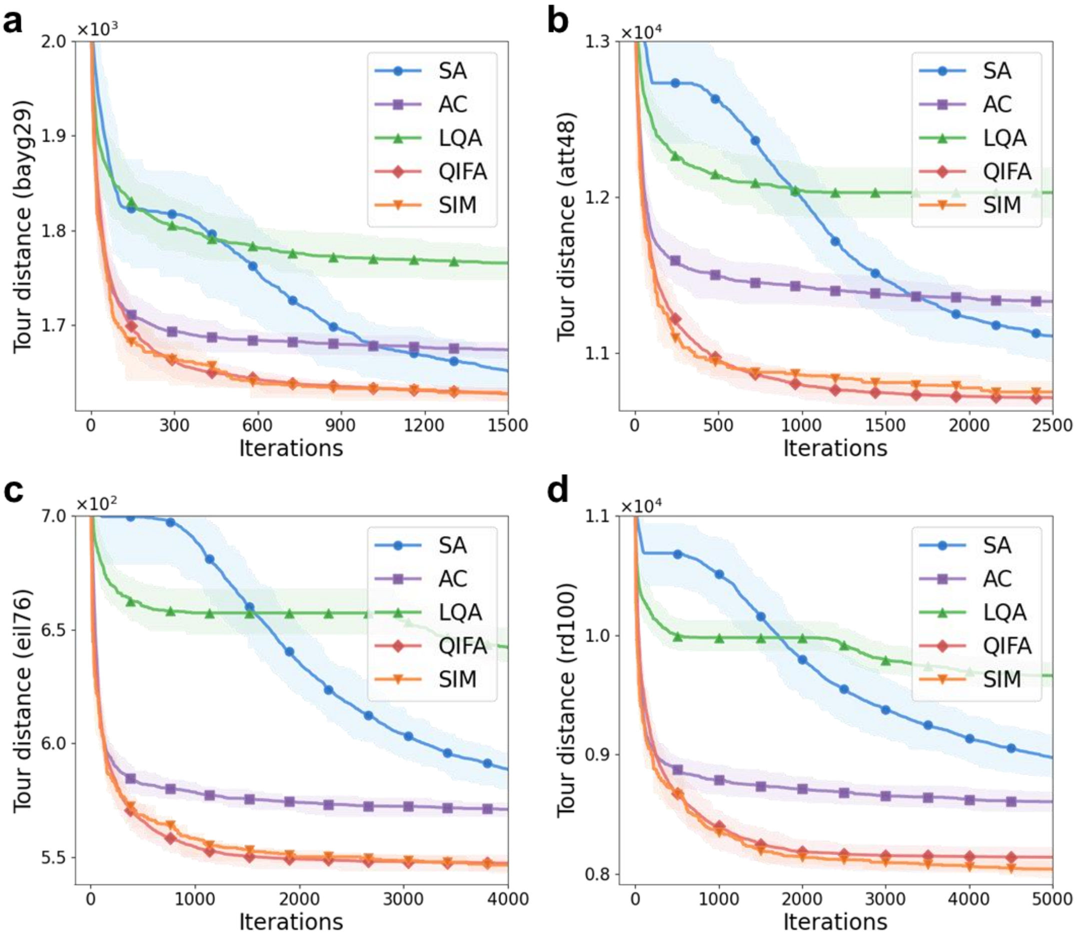

Fig. S4. Comparison of simulation results and algorithmic performances on TSPLIB benchmarks. a, bayg29. **b,** att48. **c,** eil76. **d,** rd100. The red and orange lines respectively depict QIFA's theoretical and circuit-simulated PEav, while blue, purple, and green lines represent SA, AC, and LQA results, all plotted against iteration count.

Supplementary Information S7: The latitude and longitude coordinates of the 20 major cities.

The latitude and longitude coordinates of the 20 major cities used in the TSP case study are listed below, formatted as City Name (Latitude, Longitude) based on WGS-84 standard:

1. London : (51.5074° N, 0.1278° W)

2. Paris: (48.8566° N, 2.3522° E)
3. Rome : (41.9028° N, 12.4964° E)
4. Madrid : (40.4168° N, 3.7038° W)
5. Sydney : (33.8688° S, 151.2093° E)
6. New York: (40.7128° N, 74.0060° W)
7. Washington D.C.: (38.9072° N, 77.0369° W)
8. Ottawa : (45.4215° N, 75.6972° W)
9. Cairo : (30.0444° N, 31.2357° E)
10. Dubai : (25.2769° N, 55.2962° E)
11. Cape Town : (33.9249° S, 18.4241° E)
12. Istanbul : (41.0082° N, 28.9784° E)
13. Rio de Janeiro : (22.9068° S, 43.1729° W)
14. Beijing : (39.9042° N, 116.4074° E)
15. Shanghai : (31.2304° N, 121.4737° E)
16. Hong Kong : (22.3193° N, 114.1694° E)
17. Singapore : (1.3521° N, 103.8198° E)
18. Tokyo : (35.6762° N, 139.6503° E)
19. Seoul : (37.5665° N, 126.9780° E)
20. Bangkok : (13.7563° N, 100.5018° E)

Supplementary Information S8: Disorder of the circuit components

Although our theoretical derivation is exact, experimental realizations are subject to errors due to component imperfections arising from manufacturing limitations. The electronic components used in our experiments—including power supplies and resistors—exhibit inherent variations that disturb the ideal circuit Hamiltonian. This disorder subsequently influences the dynamic evolution of the circuit.

As shown in Fig. S5, we systematically evaluate the impact of component tolerance on solution quality by simulating the QIFA circuit under resistance tolerances (tol) ranging from 0% to 5%. The tour length consistently decreases with iterations under all tolerance settings

and eventually stabilizes, confirming the general robustness of the analog implementation. Notably, under the high-precision resistors (tol = 1%) used in our actual PCB experiments, the circuit achieves a final tour length closely matching the theoretical value (tol = 0), demonstrating that high-precision components effectively preserve the intended circuit Laplacian and computational accuracy. These results validate the practical feasibility of implementing QIFA with commercially available high-precision components while also quantifying the performance degradation that may occur under higher tolerance conditions.

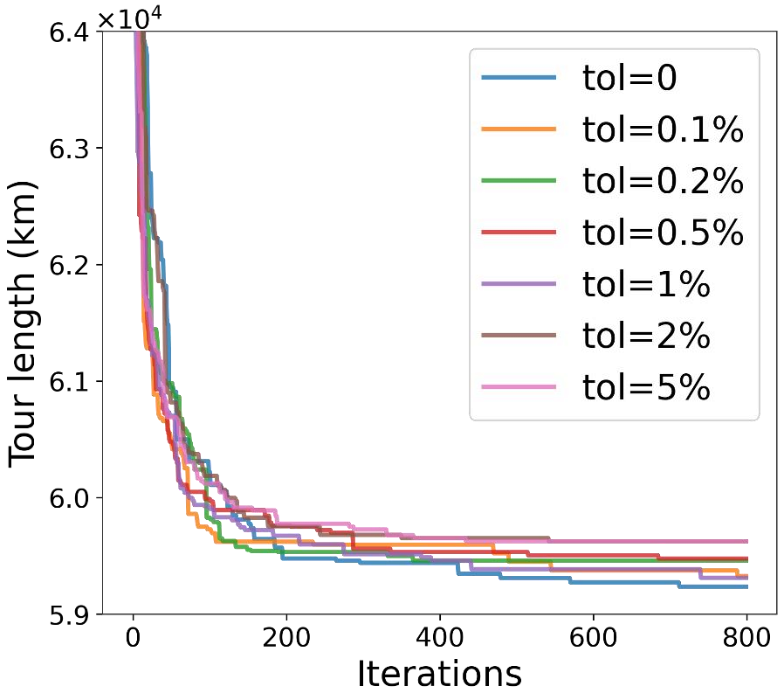

Fig. S5. Simulation results for disordered circuits. The blue, orange, green, red, purple, brown, and pink solid line show the results of resistors with tolerance 0 (ideal), 0.1%, 0.2%, 0.5%, 1%, 2% and 5%, respectively. The legend 'tol' means tolerance.

Supplementary Information S9. The QIFA theory for Knapsack Problem.

The Knapsack Problem (KP) is a canonical NP-hard constrained combinatorial optimization problem. Given a set of n items, each with a value v_i and a weight w_i, and a knapsack with a maximum weight capacity W, the problem is to select a subset of items that maximizes the total value without exceeding the weight capacity. Mathematically, the KP can be formulated as follows:

$$\max \sum_i x_i v_i \tag{S1}$$

$$\text{s.t.} \qquad \sum_i^n x_i w_i \leq C \tag{S2}$$

Here, the binary decision variable $x_i = 1$ indicates that item i is selected, and $x_i = 0$ otherwise. The inequality constraint S-2 is the capacity constraint. A standard technique involves transforming this inequality constraint into an equality by introducing a series of auxiliary binary variables $x_{n+j} \in \{0,1\}$ where $j = 0,1, \ldots, m$, with the corresponding weights $w_{n+j} = 2^j$ and values $v_{n+j} = 0$. This reformulation ensures that a flexible total weight can be represented, converting the constraint to:

$$\sum_i^{n+m} x_i w_i = W \tag{S3}$$

To integrate the KP into the QIFA framework, we first map it to the ground state search of an Ising Hamiltonian. The target Hamiltonian Hz for the KP is formulated as:

$$H_z^{KP} =- \sum_i^n v_i \frac{\sigma_i^z + 1}{2} + D\left(\sum_i^{n+m} w_i \frac{\sigma_i^z + 1}{2} - W\right)^2 \tag{S4}$$

The parameter D is a sufficiently large penalty coefficient that enforces the equality constraint. The ground state of H_z corresponds to the optimal feasible solution of the original KP.

In QIFA for KP, the time-dependent Hamiltonian is

$$H^{KP}(t) = t\gamma H_z^{KP} - (1-t) H_x^{KP} \tag{S5}$$

We introduce analog variables $\boldsymbol{\theta} = [\theta_1, \theta_2, \ldots, \theta_i, \ldots, \theta_{n+m}]^T$, where $\theta_i \in [-\frac{\pi}{2}, \frac{\pi}{2}]$ represents the orientation of the i-th spin. The time-dependent cost function corresponds to the quadratic form of $H^{KP}(t)$ with the vector $\boldsymbol{S_\theta}$:

$$C(t, \boldsymbol{\theta}) = \boldsymbol{S_\theta^T} H^{KP}(t) \boldsymbol{S_\theta} \tag{S6}$$

The partial derivative of the cost function with respect to parameter θ_i is given by:

$$\frac{\partial C(t, \boldsymbol{\theta})}{\partial \theta_i} = t\gamma \left(\frac{D w_i}{2}\left[\sum_{l \neq i} w_l (z_l + 1) - W\right] - \frac{v_i}{2}\right) x_{ij} + (1-t) z_{ij} \tag{S7}$$

where $\boldsymbol{z} = [sin\theta_1, sin\theta_2, \ldots sin\theta_n]^T$ and $\boldsymbol{x} = [cos\theta_1, cos\theta_2, \ldots cos\theta_n]^T$.

To obtain constraint-satisfying trial solutions $\boldsymbol{p}_t$ from $\boldsymbol{\theta}$, the constrained projection strategy is employed as follows: Let Y denote the set of item indices whose weight does not

exceed the remaining capacity of the knapsack. The probability P_i for the i-th item to be chosen in the solution is determined by a roulette wheel selection strategy, defined as:

$$P_i = \begin{cases} \frac{(1+z_i)^2\left(\frac{v_i}{w_i}\right)^\beta}{\sum_{i\in Y}(1+z_i)^2\left(\frac{v_i}{w_i}\right)^\beta}, & i \in Y \\ 0, & i \notin Y \end{cases} \tag{S8}$$

where β is a heuristic factor that adjusts the importance of value-to-weight ratio. In this roulette wheel selection, items whose weight exceeds the remaining capacity are excluded from the selection process, thus ensuring that the final selected item set satisfies the weight constraint.

This projection mechanism integrates seamlessly into the dynamic evolution of QIFA, where each update to the system state during the optimization process maintains feasibility by enforcing this constraint through the probabilistic selection mechanism.

Supplementary Information S10. The QIFA results for KP.

We evaluate the performance of QIFA on 100 instances of the KP, each containing 50 items with strongly correlated weights and values, from the kplib repository on GitHub. To ensure statistical robustness, each instance was solved through 1000 independent runs.

Fig. S6 shows the relationship between PEav and the number of iterations for both QIFA and SA. As seen in the plot, QIFA exhibits significantly faster convergence, with PEav approaching the optimal solution more quickly than SA. Specifically, QIFA shows near-optimal solutions after a relatively small number of iterations, while SA continues to converge more slowly, taking more iterations to achieve similar solution quality.

These results highlight QIFA's ability to find high-quality solutions in fewer iterations, emphasizing its efficiency in solving combinatorial optimization problems like KP. They demonstrate that QIFA is not specialized for TSP but rather a general-purpose optimization tool, capable of tackling a wide range of combinatorial problems. This further establishes QIFA as a versatile, scalable solution for NP-hard constrained optimization problems.

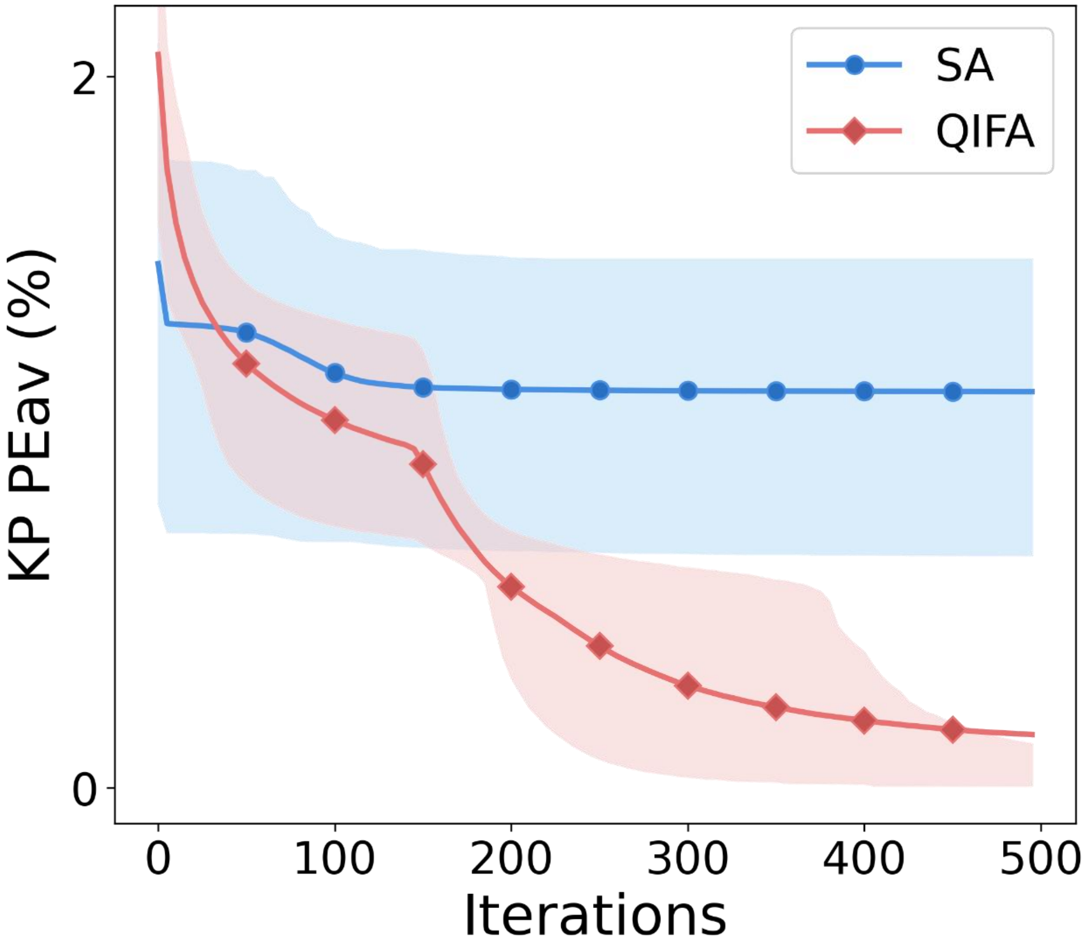

Fig. S6. Convergence comparison of QIFA and SA on KP. The figure shows the evolution of the PEav with respect to the number of iterations, averaged over 100 KP instances with 50 items from the Kplib dataset. QIFA consistently achieves lower PEav and faster convergence compared with SA. The solid lines represent the mean performance over 1000 independent runs per instance, while the shaded regions indicate the standard deviation, reflecting the statistical variability of the solutions.

[1] https://doi.org/10.1038/s42256-022-00468-6.
